# FunEball: an emerging inclusive sport to enhance engagement and fundamental movement skills in physical education

**DOI:** 10.3389/fpsyg.2026.1760902

**Published:** 2026-01-30

**Authors:** Qiannan Liu, Qi Dong, Qun Fang, Eungsoo Oh, Mingyuan Jia, Duo Yang

**Affiliations:** 1 School of Physical Education, Qingdao University, Qingdao, China; 2 Department of Physical Education, College of Sport Sciences, Dong-A University, Busan, Republic of Korea; 3 Basic Teaching Center Sports Department, Ocean University of China, Qingdao, China

**Keywords:** fundamental movement skills, FunEball, inclusive physical education, physical literacy, sport participation

## 1 Introduction

In recent years, researchers have increasingly recognized that schools play a crucial and effective role in promoting the health of children and adolescents. Through physical education (PE), physical activities, and organized sports, schools provide opportunities to improve students' physical activity levels, which can extend into adulthood (Morton et al., 2016; Love et al., 2019; Sevil et al., 2019; World Health Organization, 2022). Moreover, more than 75% of countries worldwide advocate PE as a fundamental educational requirement (Hardman et al., 2014; Shape America, 2016). Numerous studies have demonstrated that PE exerts positive effects on students' physical and mental health, cognitive and academic performance, social development, moral character, and cultural engagement, positioning PE as a cornerstone of 21st-century communities (Donnelly et al., 2016; Johnson and Turner, 2016; Watson et al., 2017; Andermo et al., 2020; Habyarimana et al., 2022). Therefore, ensuring full participation of students in PE classes is essential.

However, previous studies have identified numerous barriers on students' participation in PE. These include the perceived difficulty of mastering motor skills (Gavin et al., 2016; Oh and Hong, 2016), limited movement competence (Martins et al., 2021), lack of enjoyment (James et al., 2018; Laird et al., 2018; Palmer-Keenan and Bair, 2019), desire for novel sports experiences (Carlin et al., 2015; Martins et al., 2018), and social exclusion of students with lower athletic ability (Beltrán-Carrillo et al., 2018; Casey et al., 2016). As a result, many students exhibit low engagement and limited physical activity in PE. This raises a critical question: how can PE teachers increase students' participation and enjoyment in PE, particularly for those with low fitness levels, limited motor skills, or disabilities?

To address this issue, it is necessary to explore innovative physical activities that foster inclusiveness, participation, and enjoyment, while promoting fundamental movement skills (FMS). The emerging sport FunEball offers such potential, a novel sport developed to engage all students regardless of their ability levels.

FunEball was invented in 2015 by Professor Eungsoo Oh in South Korea. It was designed upon three core principles: cooperation, maximum participation, and fun. Drawing inspiration from volleyball and footvolley, FunEball simplifies skill requirements and modifies rules to enhance accessibility. Players can use any body part—hands, feet, thighs, or head—to strike or catch the ball, enabling diverse movements and reducing technical barriers. The slogan of FunEball— “A New Inclusive Physical Activity for All” — reflects its inclusive spirit, offering an ideal form of physical activity for individuals with moderate or lower physical fitness, including people with disabilities.

Fundamental movement skills, such as running, jumping, and throwing, are the foundation for lifelong physical activity (Logan et al., 2018). Proficiency in object control skills (catching, passing, kicking) is positively associated with physical fitness, confidence, executive function, and healthy lifestyle development (Bremer and Cairney, 2018; Capio et al., 2015; Lubans et al., 2010). Moreover, children with higher object control competence are more likely to engage successfully in physical activities (Lopes et al., 2011; Vandorpe et al., 2012). The development of endurance, strength, power, balance, and flexibility remains essential for youth (van Baak et al., 2021). As such, developing these foundational abilities through inclusive sports like FunEball is essential for overall youth development.

Compared with traditional sports, FunEball features simple rules, lower physical demands, easy to learn, and suitable for participants of all ages and ability levels. Its low competitiveness minimizes injury risks and psychological pressure, providing an accessible opportunity for less-skilled youth to enjoy physical activity. Previous research has demonstrated that implementing FunEball in university PE programs is both feasible and effective (Chung and Oh, 2016), suggesting strong potential for application in primary and secondary schools.

After continuous refinement, FunEball was first introduced internationally at Marshall University and the West Virginia Association for Health, Physical Education, Recreation, and Dance Conference in 2018. It has since been implemented in educational institutions across China, Korea, the United States, Thailand, the Philippines, Japan, and Singapore, receiving positive feedback from both teachers and students. Its net-based sport design integrates both competitiveness and technical skill, making it equally suitable for students in Western countries and in Asian contexts. This multidimensional adaptability constitutes an important foundation for the effective global promotion of FunEball. Previous research supports its high levels of participant satisfaction and inclusiveness, making it a promising innovation in PE (Mak and Oh, 2019; Chung and Oh, 2016). Building upon this foundation, this paper proposes integrating FunEball into PE to enhance students' fundamental movement skills and overall physical activity.

## 2 Potentials for developing an inclusive physical education class

The name FunEball originates from a combination of “Foot & Hand & Head + Fun” and “Everybody” and “Easy,” reflecting the sport's inclusive design and emphasis on enjoyment. Professor Oh developed the concept after observing that traditional volleyball and footvolley were less accessible to women and children, who found it challenging to use feet and heads effectively. Therefore, FunEball allows the use of all body parts, creating a more inclusive and enjoyable experience for participants of all genders and ages. Its core philosophy centers on cooperation, full participation, and fun (Korea Fhun-E Ball Association, 2015).

**Figure 1:**
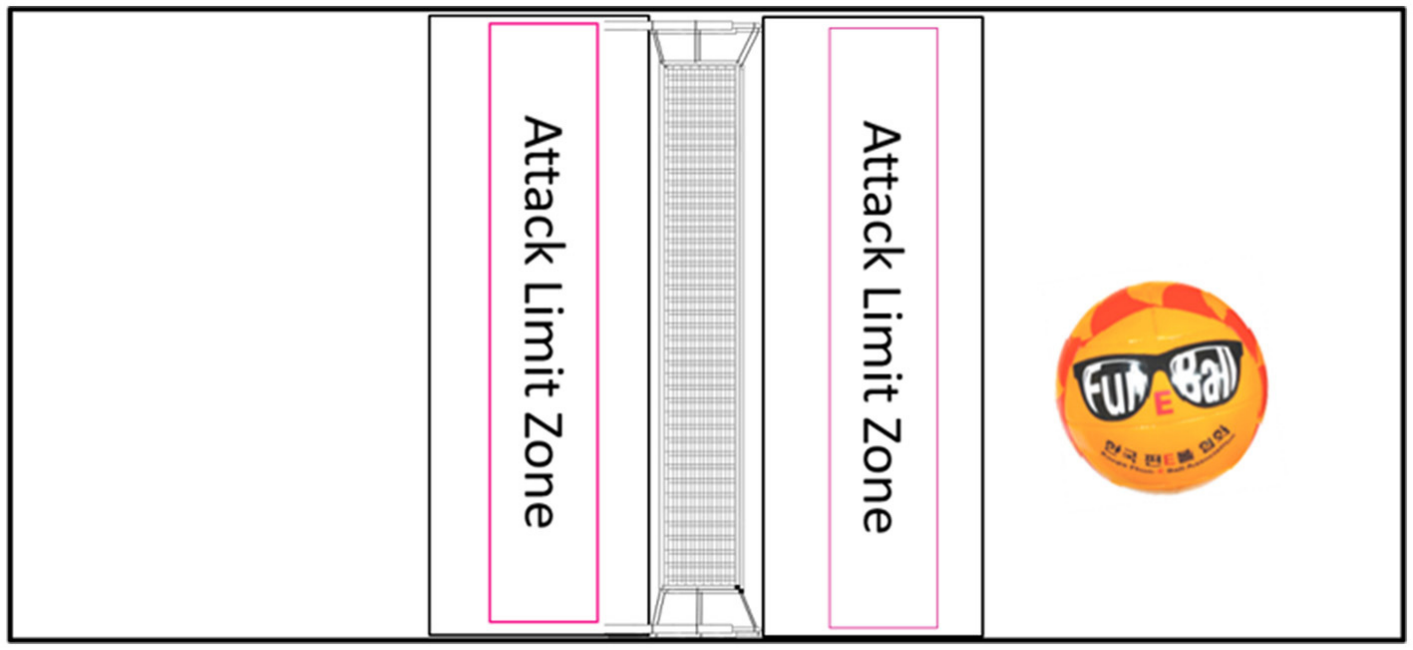
The field and ball of FunEball.

FunEball is a net-based team sport typically played by 4–9 players per team, involving passing, catching, and throwing. The field and ball for FunEball are shown in Figure 1.

The main rules include:

Serving: The game begins with a serve from behind the end line, using any throwing style (one-hand, two-hand, overhand, underhand, standing, or running throws). The receiving team must catch the ball after no more than one bounce, or they lose a point.

Passing: The offensive team must pass the ball within their side at least three times but not more than seven times before sending it over the net. Players may pass while standing, walking, or running, but must release the ball within five steps. Dropped passes result in a turnover.

Attack: Attacks must be made behind the attack limit zone; attacks cannot be launched inside this area. However, players are free to enter this zone to receive and pass the ball. And players can throw the ball into the opponent's court but not directly at players. After scoring, teams rotate positions clockwise.

Scoring: A successful serve scores 1 point; attacks completed after 3–6 passes score 2 points, and those after 7 passes score 3 points (limited to two occurrences per set). Failed attacks award 1 point to the opposing team (Korea Fhun-E Ball Association, 2015).

Innovative sports activities can address the limitations inherent in traditional sports programs, and the integration of diverse emerging sports is essential for effective physical education instruction (Oh, 2016). As introduced earlier, FunEball is an ideal and comprehensive physical activity that promotes the development of multiple motor abilities through diverse body movements. Its technical variability makes it particularly suitable for incorporation into physical education classes for children and adolescents. This sport facilitates the overall development of students' fundamental movement skills (FMS) and physical capacities, encompassing locomotor skills such as running, jumping, and dodging; object control skills such as throwing, kicking, catching, and striking; as well as stability skills related to balance and body control (Goodway et al., 2019). Moreover, FunEball enhances various physical fitness components, including strength, power, agility, reaction speed, balance, coordination, and endurance, while simultaneously fostering teamwork and cooperative awareness among students.

FunEBall is an inclusive sport which combines rules and techniques of footvolley and volleyball. By lowering the net height, restricting attack zones, allowing flexible serving styles, and using a lightweight hybrid ball, it reduces skill difficulty and enhances accessibility. The rotation of serving players prevents domination by highly skilled individuals, encouraging equitable participation (Oh et al., 2015). Additionally, allowing up to seven touches per rally decreases pressure and increases opportunities for teamwork compared with volleyball and footvolley, where only three touches are permitted (Won and Soon, 2012). This design ensures that every participant can engage meaningfully, making it an ideal sport for inclusive PE settings.

FunEBall is easy to organize, which is another prominent advantage in application. The sport requires minimal equipment—a hybrid ball combining properties of volleyball and soccer balls and a net—and can be played indoors or outdoors on courts of adjustable size. Net height, court dimensions, and team size can be adapted based on participants' age, skill, or fitness level. This adaptability makes FunEball particularly suitable for inclusive PE instruction.

However, existing studies have largely focused on theoretical or descriptive approaches, and the lack of experimental data constrains the quantitative evaluation of the effects of FunEball, thereby constituting a major limitation of the current body of research.

## 3 PE class design based on FunEball

The rules of FunEball are highly simple and flexible, allowing for adjustments based on learners' age, physical fitness levels, and instructional objectives. For example, modifications may include enlarging or reducing the playing area, determining whether kicking is permitted, adjusting offensive time limits, or altering team size. Such adaptability is a key factor contributing to the successful integration of FunEball into physical education classes.

First, students can be divided into small teams (e.g., 4v4, 5v5, or 6v6) according to students' age, physical condition to manipulate physical intensity. Smaller-sided games elicit higher heart rates, blood lactate levels, and perceived exertion (Little and Williams, 2007), making them ideal for endurance training, and suggesting effective engagement even with limited participants.

Similarly, adjusting court size can regulate physical load. Reducing the playing area lowers running distance and intensity, making it suitable for younger or less-skilled students. Besides, the ratio of play-to-rest time can also be altered to control physiological demands: higher ratios for endurance training, and lower ones for skill and tactical learning.

Also, teachers can further modify the number and duration of passes to improve agility and teamwork. For instance, requiring five passes per rally with a five-second holding limit promotes quick decision-making and coordination. Beginners may be given longer possession times and more passes, with difficulty increasing progressively. Additional rules can incorporate kicking or foot passes to enhance coordination, control, and reaction skills.

This study has taken into account the challenges that may arise during the implementation of FunEball. Given that FunEball requires students to acquire a wide range of motor skills, object control skills, and stability skills, the demands of learning and mastering numerous movements and techniques pose a substantial challenge to students' learning capacity. From the teachers' perspective, effectively organizing and delivering instruction within limited class time so as to facilitate efficient skill acquisition represents another significant challenge.

To address these challenges, the present study proposes several targeted recommendations for PE class design. First, students within the same class should be grouped according to their motor competence levels, thereby facilitating peer interaction, mutual support, and more effective skill acquisition. Second, instructional content should be differentiated according to students' age-related characteristics, with skill learning arranged in a progressive and developmentally appropriate manner across grade levels. When designing lesson plans, teachers should adopt a structured and systematic approach. For example, students in lower grades should primarily focus on developing fundamental skills such as running, jumping, throwing, and catching. Students in middle grades can gradually transition to more complex skills, including dodging, passing, and kicking. For students in higher grades, instruction should not only aim to refine previously acquired skills but also place greater emphasis on balance and body control, as well as the development of tactical awareness and decision-making abilities. Finally, competition rules should be designed flexibly. Game difficulty can be adjusted according to students' age and motor proficiency levels. For instance, for younger students or those with lower motor competence, kicking may be prohibited during games, or allowances such as permitting one dropped ball per round can be introduced to reduce task difficulty and enhance participation.

## 4 Conclusion

This study introduces FunEball, a novel inclusive sport now being promoted in China. The current study proposed an opinion on the promising effects of integrating FunEball into PE classes to enhance students' fundamental movement skills, physical activity levels, and enjoyment of participation. Characterized by fun, inclusivity, flexibility, and simple rules, FunEball provides an engaging environment where students of all abilities can participate meaningfully. By adjusting court size, team numbers, and rule complexity, PE teachers can tailor FunEball to diverse educational contexts, fostering not only motor skill development and physical fitness but also teamwork and enjoyment. As such, FunEball represents a promising and innovative approach to inclusive physical education, aligning with the global movement toward active, equitable, and engaging learning environments for all students. In the future, additional experimental and comparative studies should be conducted to generate empirical evidence, thereby enabling a more precise evaluation of the specific effects of FunEball on students across different educational stages.
